# Structural characterization and effect on diabetes mellitus with ulcerative colitis of polysaccharide from *Chimonanthus salicifolius*

**DOI:** 10.3389/fnut.2026.1934103

**Published:** 2026-09-21

**Authors:** Xiaoqin Zhang, Shuang Liu, Bingfeng Lin, Jiale Mao, Tao Li, Songquan Wu, Xin Fu, Hongliang Zhang, Houxing Lei, Nani Wang

**Affiliations:** 1 Department of Pharmacy, Lishui Hospital of Traditional Chinese Medicine, Lishui, China; 2 Zhejiang Provincial Key Laboratory of She Medicine Inheritance, Innovation, Development and Application of Traditional Chinese Medicine, Lishui, China; 3 Department of Medicine, Zhejiang Academy of Traditional Chinese Medicine, Hangzhou, China; 4 School of Medicine, Lishui University, Lishui, China; 5 Lishui She Medicine Inheritance, Innovation, Development and Application Key Laboratory of Traditional Chinese Medicine, Lishui, China

**Keywords:** *Chimonanthus salicifolius*, diabetes mellitus, polysaccharide, structural characterization, ulcerative colitis

## Abstract

**Introduction:**

Shiliang tea, derived from the leaves of *Chimonanthus salicifolius*, has traditionally been used to manage gastrointestinal disorders. This study aimed to isolate and characterize a polysaccharide from *Chimonanthus salicifolius* (CSP) and evaluate its therapeutic effects in a murine model of diabetes mellitus‑associated colitis.

**Methods:**

The structural features of CSP were determined using gel permeation chromatography, gas chromatography–mass spectrometry, and nuclear magnetic resonance spectroscopy. Following oral administration of CSP, colonic tissues from diabetic mice were collected for histological examination. Concentrations of short‑chain fatty acids (SCFAs) in colonic contents were measured by gas chromatography.

**Results:**

A polysaccharide (CSP) with an approximate molecular mass of 574.7 kDa was isolated from Chimonanthus salicifolius. Methylation analysis revealed that CSP comprises ten distinct sugar residues, while NMR spectra showed the presence of terminal α-arabinofuranosyl (T-α-Araf), 1,5- and 1,3,5-linked α-Araf, terminal β-galactopyranosyl (T-β-Galp), 1,3,6-, 1,4,6-, 1,4-, and 1,3-substituted β-Galp, as well as 1,4- and 1,3-linked α-glucopyranosyl (α-Glcp) residues in CSP. CSP administration markedly attenuated hyperglycemia and impeded the progression of ulcerative colitis in diabetic mice. CSP significantly suppressed pro inflammatory cytokine production, predominantly via blockade of NF‑κB signaling cascade. CSP reinforced intestinal epithelial barrier function through upregulation of tight junction associated proteins, including claudin 1, occludin, and zonula occludens 1, and concurrently elevated the concentrations of SCFAs within the colonic lumen.

**Conclusion:**

These results indicate that CSP is a bioactive polysaccharide with demonstrable biological activity in animal models, warranting further investigation as a potential therapeutic agent for diabetic colitis.

## 1 Introduction

Shiliang tea, an excellent functional green tea, is a healthy drink popular among traditional families of She nationality, and is recognized for its antioxidative, antimicrobial, and anti-inflammatory activities (1). *Chimonanthus salicifolius* and *Chimonanthus zhejiangensis*, are the botanical sources of Shiliang tea, the tea-making product prepared from their dried leaves. The product has gradually become popular largely because of its efficacy in the management of gastritis, ulcerative colitis (UC), and other gastrointestinal disorders. Modern phytochemical studies have identified polysaccharides, volatile oils, flavonoids, and phenolic acids as their major bioactive constituents (2). Among these, polysaccharides have demonstrated significant therapeutic potential, particularly in the management of diabetes, colonic diseases, cancer, and cardiovascular conditions (3, 4). However, the polysaccharides from *Chimonanthus salicifolius* have not been studied.

UC, a prevalent subtype of inflammatory bowel disease (IBD), is a long-term inflammatory disorder influencing the digestive tract, typically marked by clinical manifestations, including abdominal pain, diarrhea, rectal bleeding, and weight loss (5, 6). Diabetes mellitus (DM), an autoimmune disorder primarily affecting children and adolescents, results from the destruction of pancreatic β-cells (7). The global incidence of DM is rising rapidly, with an annual increase estimated at 2–5% (8). A growing body of evidence has revealed a strong association between IBD and DM (9–11). Common treatments for UC, such as corticosteroids, can induce hyperglycemia, posing particular risks for patients with pre-existing diabetes (12). Moreover, complications of UC, including dehydration, electrolyte disturbances, sepsis, and reliance on parenteral nutrition—can increase the likelihood of hyperosmolar hyperglycemia and diabetic ketoacidosi (13). These clinical challenges highlight the urgent need for novel therapeutic agents capable of simultaneously addressing both UC and DM.

Previous studies have demonstrated that *Chimonanthus salicifolius* extract alleviates colitis induced by dextran sulfate sodium and influence gut microbiota composition in mice (14, 15). Polysaccharides sourced from traditional Chinese medicine have been reported to exert therapeutic effects on both colitis and DM through different signaling pathways (16, 17). Therefore, we hypothesized that the polysaccharides from *Chimonanthus salicifolius* may possess dual efficacy in alleviating DM complicated by UC.

In the present study, a predominant polysaccharide fraction *Chimonanthus salicifolius* (CSP) was isolated and purified. The structural characteristics of CSP were elucidated utilizing high-performance liquid chromatography (HPLC), heteronuclear multiple bond correlation (HMBC), correlation spectroscopy (COSY), nuclear magnetic resonance (NMR), and methylation analysis. To establish a mouse model of DM with concurrent UC, mice were intraperitoneally injected with alloxan monohydrate, and CSP was used to investigate its effects. This study aims to preliminarily analyze the structure of CSP and observe whether it simultaneously affects glucose homeostasis and intestinal inflammation in the DM and UC combined model.

## 2 Materials and methods

### 2.1 Materials

*Chimonanthus salicifolius* was collected from the LISHAN mountains in Lishui, Zhejiang Province. The plant specimen was authenticated as *Chimonanthus salicifolius* by Chief Pharmacist Lin Na from Lishui Hospital of Traditional Chinese Medicine (Figure 1A). A voucher specimen (No. LYLM-1) has been deposited in the Specimen Museum of Lishui Hospital of Traditional Chinese Medicine. The TSKgel used for column chromatography was purchased from Tosoh Corporation (Japan). Antibodies were supplied by Boster Biological Technology Co., Ltd. (Wuhan, China). SYBR Green dye was supplied by ABclonal Biotech Co., Ltd. (Wuhan, China). Dextran and Dextran from *Leuconostoc mesenteroides* were obtained through Sigma-Aldrich (USA).

**Figure 1:**
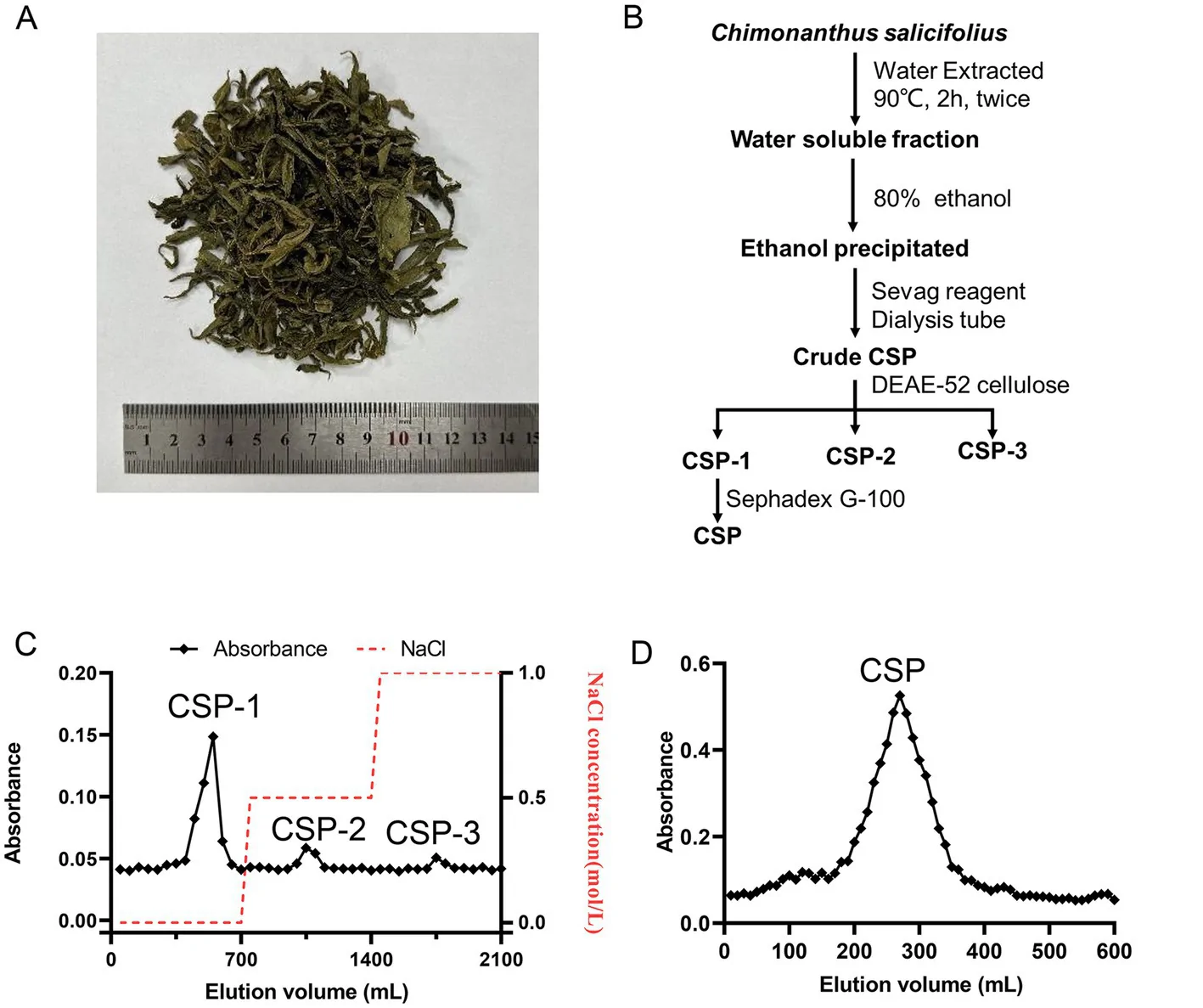
Extraction and fractionation of CSP from *Chimonanthus salicifolius*. **(A)** Dried leaves of *Chimonanthus salicifolius*, **(B)** Extraction and fractionation scheme of polysaccharides, **(C)** Elution curve of CSP on DEAE-52 column, **(D)** Elution curve of CSP on Sephadex G-100 column.

### 2.2 Polysaccharides extraction and purification

Dried *Chimonanthus salicifolius* leaves (1 kg) were powdered and extracted twice with distilled water (1:10, w/v) for 1 h each. The pooled aqueous extracts were passed through a 200-mesh filter, concentrated under reduced pressure, and precipitated with four volumes of ethanol. After standing at 4 °C overnight, the resulting precipitate was collected and redissolved in water. The solution was deproteinated with Sevag reagent (trichloromethane:n-butanol, 4:1, v/v), followed by dialysis (molecular weight (Mw) cutoff: 3500 Da) against flowing water for 3 d. The remaining solution in the dialysis tube was collected, concentrated, and precipitated using four volumes of anhydrous ethanol at 4 °C for 12 h. The residue was lyophilized to obtain crude polysaccharides from *Chimonanthus salicifolius* (Crude CSP).

Crude CSP was then dissolved (20 mg/mL) in ultrapure water and subjected a DEAE-52 cellulose column (2.5 × 45 cm), followed by elution with ultrapure water, along with 0.5 mol/L NaCl, or 1.0 mol/L NaCl, resulting in three polysaccharide fractions, CSP-1, CSP-2, and CSP-3, respectively. CSP-1 was further purified using a Sephadex G-100 column (1.5 × 100 cm), resulting in one homogeneous polysaccharide fractions named purified *Chimonanthus salicifolius* (CSP). The purified CSP was lyophilized and stored at 4 °C.

### 2.3 Chemical compositions

Quantification of total carbohydrates in CSP was conducted via the phenol–sulfuric acid colorimetric assay with glucose standards at 0.02, 0.04, 0.06, 0.08, and 0.10 mg/mL. Standard solutions (0.4 mL) were mixed with 5% phenol (0.2 mL) and concentrated H_2_SO_4_ (2.0 mL), and absorbance at 490 nm was monitored to establish a calibration curve for quantification. UV-2450 ultraviolet–visible (UV) spectrophotometer was used to scan the CSP solution at the wavelength range of 190–700 nm to detect whether there was any significant absorption peak of nucleic acid and protein conjugate at 260 nm and 280 nm, and protein content was quantified by BCA assay. The uronic acid content was determined by the carbazole-sulfuric acid method using glucuronic acid as standard.

### 2.4 Molecular weight

Mw of CSP was determined using an 1,260 infinity II high-performance liquid chromatography equipped with an evaporative light scattering detector (Agilent Technologies, USA) and a TSKgel G5000PWXL column (7.8 × 300 mm; Tosoh, Japan). The sample (1.0 mg/mL) was eluted using water as the mobile phase at a 0.6 mL/min flow rate. The injection volume was 10 μL. Mw of polysaccharide was calculated by comparison with retention time-Mw curve derived from a series of standard dextrans (Mw = 1.0, 5.0, 25.0, 150.0, 410.0, and 670.0 kDa, respectively; Sigma-Aldrich).

### 2.5 Monosaccharide composition analysis

The monosaccharide composition of CSP was analyzed following hydrolysis with 3 mol/L trifluoroacetic acid at 120 °C for 3 h. After nitrogen-assisted drying, the residue was dissolved in deionized water. Monosaccharide profiling was performed by HPLC using a Dionex Carbopac™ PA20 column (3 mm × 150 mm, Thermo Scientific) coupled with electrochemical detection. Mobile phases comprised (A) water, (B) 15 mM NaOH, and (C) 15 mM NaOH containing 100 mM sodium acetate. The injection volume was 5 μL, with a flow rate of 0.3 mL/min at 30 °C. Gradient elution was applied as follows: 0–18 min (98.8:1.2:0), 18–30 min (50:50:0), 30.1–46 min (0:0:100), 46.1–50 min (0:100:0), and 50.1–80 min (98.8:1.2:0).

### 2.6 Methylation analysis

Methylation analysis was performed based on established protocols. The resulting partially methylated alditol acetates were subjected to GC–MS for glycosidic linkage determination using spectral comparisons with known standards. Analyses were conducted on a Shimadzu GCMS-QP2010 system fitted with an RXI-5 SIL MS capillary column (30 m × 0.25 mm i.d., 0.25 μm film thickness). The oven temperature was initially set at 120 °C and ramped to 250 °C at 3 °C/min, with the final temperature held for 5 min. The injector and mass spectrometer interface were both maintained at 250 °C. Helium served as the carrier gas at a constant 1.0 mL/min flow rate.

### 2.7 NMR spectroscopic analysis

The appropriate amount of CSP was dissolved in D_2_O, and the ^1^H-NMRM, ^1^C-NMR, HMBC, HSQC and COSY spectra were used to determine the characteristic of CSP at 25 °C.

### 2.8 Animals

Male C57BL/6 mice weighing 20 ± 3 g were obtained from Hangzhou Medical College. All experimental protocols were carried out in strict accordance with the guidelines outlined in the NIH Guide for the Care and Use of Laboratory Animals and received ethical approval from the Institutional Animal Care and Use Committee of the Zhejiang Academy of Traditional Chinese Medicine (Protocol: 2022–031). Animals were housed under standardized conditions (25 °C, 60 ± 5% relative humidity) with ad libitum access to food and water. A 7-day acclimatization period was provided prior to the initiation of experimental procedures.

### 2.9 DM experimental model and grouping

The experimental design is shown in Figure 2A. A single intraperitoneal dose of alloxan monohydrate (180 mg/kg) was administered to induce the DM model (7). After 72 h, fasting blood glucose (FBG) was measured following an 8-h fast. Mice exhibiting FBG values exceeding 11.1 mmol/L were considered to have successfully developed diabetes. These mice were randomly allocated into four cohorts (*n* = 5 per group) (7): control group with saline, positive control group with 100 mg/kg/d metformin hydrochloride (METH) (18), CSP treatment group with 50 mg/kg/d, model group with saline. All groups except the control group were injected with alloxan to induce DM. All animal procedures, including body weight assessments, sample collection, and biochemical analyses, were conducted under blinded conditions to reduce observer bias. Body weights were recorded weekly throughout the experimental period. After 4 weeks, animals were fasted for 12 h, anesthetized via inhalation of 3% isoflurane and bled from the orbital sinus, and then euthanized by cervical dislocation.

**Figure 2:**
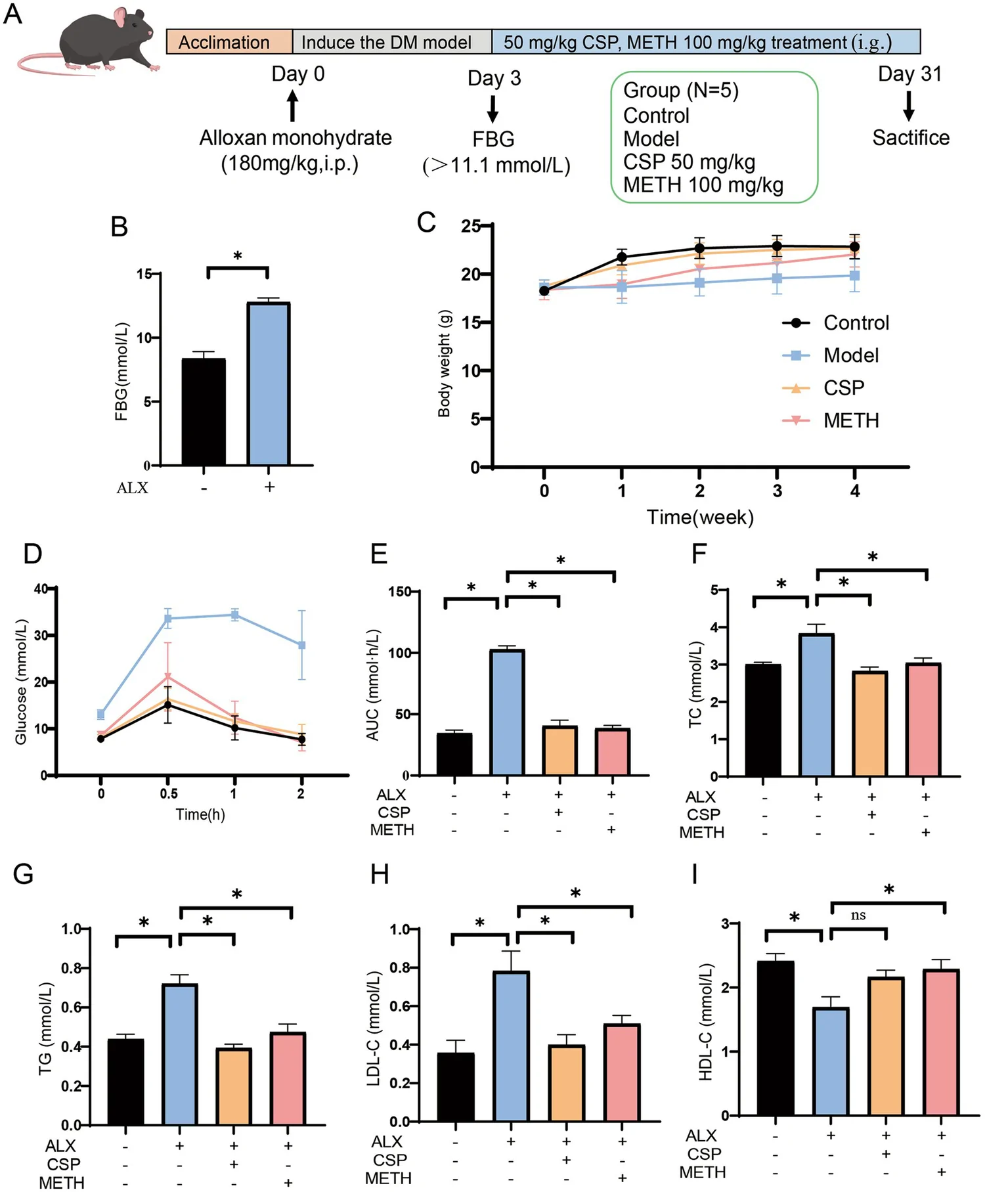
CSP inhibits the progression of diabetes in DM mice. **(A)** Timeline of animal experiments (mouse image created with BioGDP.com). **(B)** Fasting blood glucose levels after alloxan (ALX) injection. **(C)** Body weight measurements during the experimental period. **(D)** CSP treatment alleviates hyperglycemia in DM mice. **(E)** CSP reduces the AUC of glucose tolerance in DM mice. Effects of CSP on serum levels of **(F)** triglycerides TG, **(G)** TC, **(H)** LDL-C, and **(I)** HDL-C in DM mice. (n = 5 per group; *P < 0.05, **P < 0.01 compared with model group, ns, no significant *P* ≥ 0.05).

### 2.10 Detection of blood glucose and blood lipid index

FBG levels were measured utilizing tail vein blood samples. Glucose tolerance was evaluated through oral glucose gavage (2.0 g/kg), followed by sequential blood glucose measurements at 0, 30, 60, and 120 min post-gavage. The area under the curve (AUC) was calculated utilizing the equation: AUC = 0.5 × (0 min) + (30 min) + 1.5 × (60 min) + (120 min) glucose values. Serum lipid profiles, including triglycerides (TG), total cholesterol (TC), high-density lipoprotein cholesterol (HDL-C), low-density lipoprotein cholesterol (LDL-C), and myeloperoxidase (MPO) levels were measured utilizing ELISA kits (Boster, China).

### 2.11 Pathological and immunohistochemical analysis

Colon specimens were initially fixed in 10% neutral-buffered formalin for 24 h, followed by a graded dehydration process and paraffin embedding. Paraffin-embedded tissues were sectioned at 5 μm thickness and subjected to hematoxylin and eosin (H&E) staining for morphological assessment. Microscopic images were captured at 400 × magnification using a light microscope. For immunohistochemical analysis, selected colon tissue samples were fixed in 4% paraformaldehyde at room temperature for 24 h and embedded in paraffin. Sections were cut at 3 μm thickness and co-stained with antibodies against TNF-α, IL-1β, IL-6, Claudin-1, and Occludin. Fluorescence signals were visualized and recorded utilizing a CCD-equipped fluorescence microscope (Olympus, Japan).

### 2.12 RT-PCR analysis

Total RNA was isolated from colon tissues utilizing the TRIzol method. cDNA was synthesized by reverse transcription, and gene expressions like TNF-α, IL-1β, IL-6, Claudin-1, Occludin, and ZO-1 were quantified utilizing RT-PCR (Supplementary Table S1). GAPDH served as the internal control, and relative gene expression was evaluated utilizing the 2^–ΔΔCt^ method.

### 2.13 Western blot analysis

Colon tissues were lysed in Western blot buffer, and total protein content was quantified with a commercial assay kit (KeyGEN Biotech, Shanghai, China). Equal amounts of protein were resolved on 10% SDS-polyacrylamide gels and electrotransferred onto polyvinylidene fluoride (PVDF) membranes. Membranes were blocked for 2 h at ambient temperature with 5% non-fat dry milk prepared in TBST buffer (5 mM Tris–HCl, 136 mM NaCl, 0.05% Tween-20, pH 7.6), followed by overnight incubation at 4 °C with specific primary antibodies diluted 1:500. After thorough washing, membranes were probed with horseradish peroxidase (HRP)-labeled secondary antibodies. Enhanced chemiluminescence (ECL) was used for protein visualization, and band quantification was conducted utilizing Image-Pro Plus software (version 6.0).

### 2.14 Targeted metabolite analysis

Short-chain fatty acids (SCFAs) in mouse colonic contents were quantified by gas chromatog raphy−mass spectrometry (GC–MS). Analyses were conducted on a Thermo Scientific TRACE-1600 gas chromatograph coupled with an ISQ-7610 mass spectrometer, fitted with a TG-WAXMS capillary column (30 m × 0.25 mm i.d., 0.25 μm film thickness). The oven temperature was initially set at 120 °C and held for 5 min, then ramped to 200 °C at 40 °C/min, with the final temperature held for 2 min. Briefly, SCFAs were extracted from 100 mg of mouse colonic contents in 1 mL of 0.1% phosphoric acid solution. The mixture was centrifuged at 12,000 g and 4 °C for 10 min to collect the supernatant. SCFAs were extracted from the supernatant with an equal volume of ethyl acetate, and concentrations of acetic acid, propionic acid, butyric acid and valeric acid, were quantified by GC–MS using an external standard calibration.

### 2.15 Statistical analysis

Data are presented as mean ± standard deviation (SD). Statistical comparisons were performed utilizing one-way ANOVA or Student’s *t*-test with GraphPad Prism 8 (GraphPad Software Inc., USA). Differences were considered significant at *p* < 0.05 or *p* < 0.01.

## 3 Results

### 3.1 CSP isolation and purification

The separation procedure is illustrated in Figure 1B. Crude polysaccharides were initially obtained from *Chimonanthus salicifolius* leaves through hot water extraction and ethanol precipitation, yielding a crude polysaccharide content of 5.31%. The crude extract was separated by a DEAE-52 anion exchange column. After elution with ultrapure water, three polysaccharide components were obtained (Figure 1C). The polysaccharide fraction CSP-1 (67.21% yield of crude polysaccharide) was further purified on a Sephadex G-100 column, and the purified polysaccharide CSP was obtained (Figure 1D, 75.68% yield of CSP-1). Figure 3A shows that CSP had a single symmetrical peak. The Mw of CSP was calculated to be 574.7 kDa. The total content of sugar in CSP was 95.62%, which was determined in accordance with the phenol-sulfuric acid method. Furthermore, there were no obvious absorption peaks at 260 nm and 280 nm in the UV spectrum (Figure 3B), which indicated that CSP had no nucleic acid and protein, and the BCA assay corroborated the absence of protein. Carbazole-sulfuric acid assay showed that there was no uronic acid in CSP. The monosaccharide composition analysis (Figure 3C) showed that CSP is composed of arabinose (Ara), galactose (gal), and glucose (Glc) at a molar ratio of 4:5:10.

**Figure 3:**
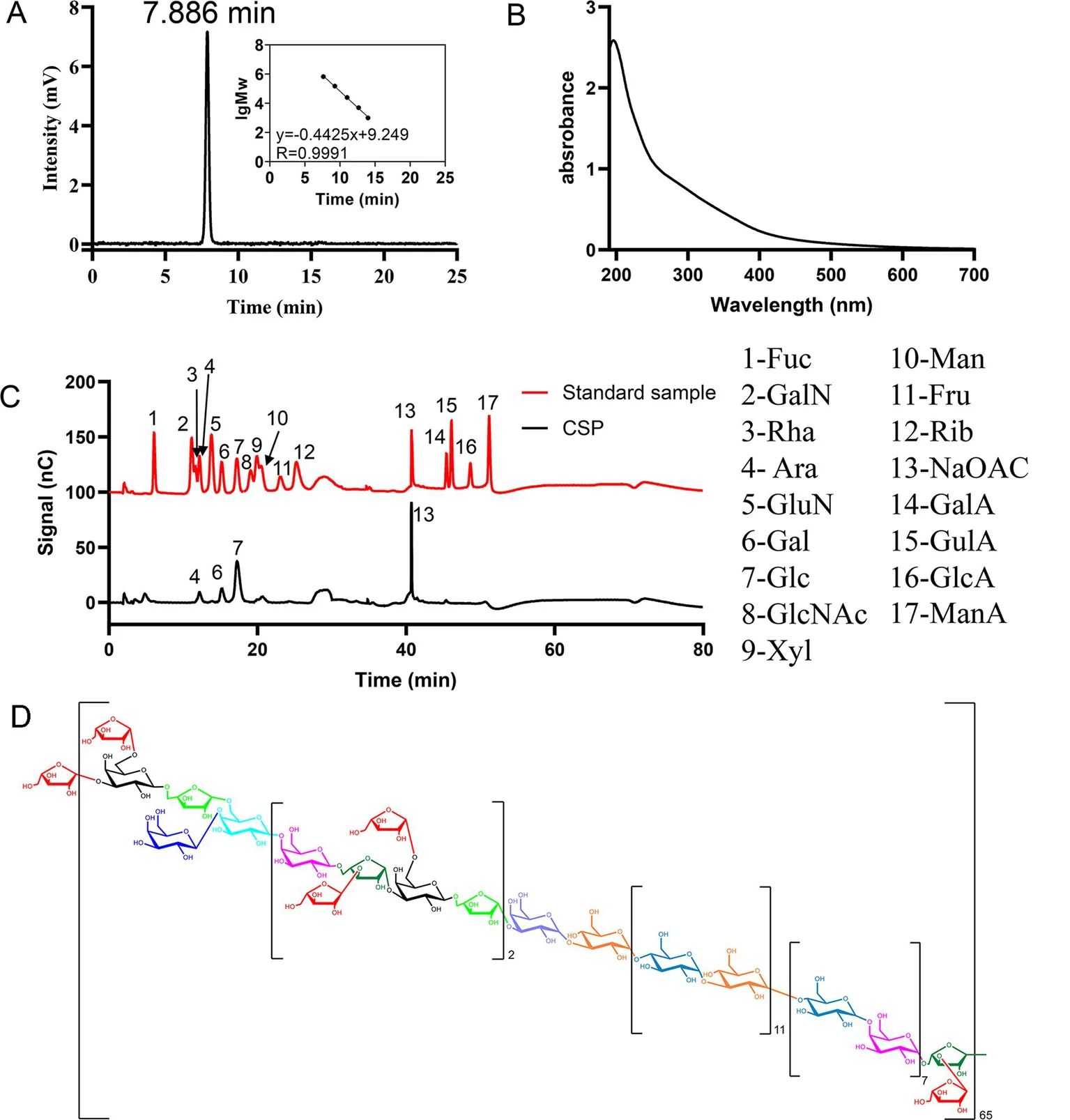
Physicochemical analysis of CSP. **(A)** Molecular weight determination of CSP. **(B)** UV spectrum of CSP. **(C)** Monosaccharide composition analysis of CSP. **(D)** Hypothesized structure of CSP.

### 3.2 Structural linkage analysis of CSP by methylation analysis

Methylation analysis revealed that CSP is composed of ten sugar residues, identified through retention times and fragmentation patterns matched against the Complex Carbohydrate Research Center (University of Georgia) spectral database (Table 1). Arabinose residues included T-Araf, 1,5-Araf, and 1,3,5-Araf; galactose was present as T-Galp, 1,3-, 1,4-, 1,3,6-, and 1,4,6-linked Galp; and glucose residues were identified as 1,4- and 1,3-linked Glcp.

**Table 1:** Methylation analysis data for CSP.

RT	Methylated sugar	Mass fragments (m/z)	Molar ratio (%)	Type of linkage
16.097	2,3,5-Me_3_-Araf	43, 71, 87, 101, 117, 129, 145, 161	12.1	Araf-(1→
21.318	2,3-Me_2_-Araf	43, 71, 87, 99, 101, 117, 129, 161, 189	6.2	→5)-Araf-(1→
25.871	2-Me_1_-Araf	43, 58, 85, 99, 117, 127, 159, 201	6.1	→3,5)-Araf-(1→
24.572	2,3,4,6-Me_4_-Galp	43, 71, 87, 101, 117, 129, 145, 161, 205	1.9	Galp-(1→
38.366	2,4-Me_2_-Galp	43, 87, 117, 129, 159, 189, 233	6.7	→3,6)-Galp-(1→
36.087	2,3-Me_2_-Galp	43, 71, 85, 87, 99, 101, 117, 127, 159, 161, 201, 261	2.2	→4,6)-Galp-(1→
28.824	2,3,6-Me_3_-Galp	43, 87, 99, 101, 113, 117, 129, 131, 161, 173, 233	15.9	→4)-Galp-(1→
29.39	2,3,6-Me_3_-Glcp	43, 87, 99, 101, 113, 117, 129, 131, 161, 173, 233	27.7	→4)-Glcp-(1→
28.438	2,4,6-Me_3_-Glcp	43, 87, 99, 101, 117, 129, 161, 173, 233	18.9	→3)-Glcp-(1→
29.761	2,4,6-Me_3_-Galp	43, 87, 99, 101, 117, 129, 161, 173, 233	2.3	→3)-Galp-(1→

### 3.3 Structural analysis of CSP

^1^H-NMR spectroscopy of CSP revealed ten anomeric proton signals within the range of 5.32–4.43 ppm (Supplementary Figure S1A), while the corresponding ^13^C-NMR spectrum displayed ten anomeric carbon signals in the region of 109.24–99.61 ppm (Supplementary Figure S1B, Table 2). These data indicated the presence of ten distinct sugar residues in the polysaccharide structure.

**Table 2:** ^1^H and ^13^C NMR chemical shift assignments of CSP (δ, ppm).

Type of linkage	H1/C1	H2/C2	H3/C3	H4/C4	H5/C5	H6/C6
A: α-L-Araf-(1→	5.15	4.13	3.86	4.05	3.64/3.86	
	109.24	81.31	76.47	84.26	60.54	
B: →5)-α-L-Araf-(1→	5	4.04	3.86	4.08	3.73/3.65	
	107.39	80.88	76.99	83.66	62.53	
C: →3,5)-α-L-Araf-(1→	5.1	4.06	3.66	3.86	3.62/3.72	
	107.49	80.75	79.81	76.65	62.91	
D: β-D-Galp-(1→	4.43	3.57	3.64	4.04	3.85	3.75
	103.03	69.91	72.28	69.85	69.99	60.45
E: →3,6)-β-D-Galp-(1→	4.55	3.59	4.04	3.92	3.86	3.81
	104.03	59.57	83.69	68.57	73.22	65.64
F: →4,6)-β-D-Galp-(1→	4.67	3.32	3.66	4.09	3.87	3.73/3.74
	100.11	72.73	72.78	86.91	73.13	66.53
G: →4)-β-D-Galp-(1→	4.7	3.47	3.74	3.56	3.67	3.59/3.75
	102.64	71.15	72.99	76.27	73.1	60.37
H: →4)-α-D-Glcp-(1→	5.32	3.56	3.87	3.56	3.86	3.67/3.78
	99.89	71.4	73.27	73.69	69.15	60.14
I: →3)-α-D-Glcp-(1→	5.29	3.54	3.67	3.58	3.33	3.63/3.74
	99.97	71.75	70.96	72.67	73.08	61.19
M: →3)-β-D-Galp-(1→	4.45	3.59	4.04	3.86	3.74	3.87
	103.3	72.19	81.7	69.42	76.43	60.7

The residues were assigned using 2D correlation analysis (COSY, Supplementary Figure S2A; HSQC, Supplementary Figure S2B). Comparison with previously published data allowed the identification of sugar residues A, B, and C as T-α-Araf, 1,5-α-Araf, and 1,3,5-α-Araf, respectively (19). Their anomeric proton signals were observed at 5.16, 5.00, and 5.10 ppm. The corresponding proton resonances for other ring positions of residues A, B, and C appeared in the range of 4.13–3.64, 4.06–3.64, and 4.06–3.61 ppm, respectively. Based on reported literature, residues D, E, F, G, and M were assigned as T-β-Galp, 1,3,6-β-Galp, 1,4,6-β-Galp, 1,4-β-Galp, and 1,3-β-Galp (20–24). Their anomeric proton signals were observed at 4.43, 4.55, 4.67, 4.70, and 4.45 ppm, corresponding to anomeric carbon chemical shifts at 103.11, 104.03, 100.11, 102.64, and 103.39 ppm, respectively. The chemical shifts for C-2 to C-5 of these residues ranged from 74.10–60.43 ppm (D), 83.81–65.40 ppm (E), 86.90–66.56 ppm (F), 76.51–60.39 ppm (G), and 81.77–60.48 ppm (M). Residues H and I were identified as 1,4-α-Glcp and 1,3-α-Glcp, respectively. COSY spectra confirmed coupling between H1 and H2 at 5.32/3.56 ppm for residue H and 5.29/3.54 ppm for residue I (25, 26). The proton and carbon chemical shifts of the ten residues are listed in Table 2.

HMBC spectra were used to further elucidate the glycosidic linkages within CSP (Supplementary Figure S3). A prominent cross-peak at 102.64/3.56 ppm was attributed to G(C1)/H(H4), confirming the linkage of C1 of 1,4-β-Galp to the C4 of 1,4-α-Glcp. Additional signals such as M(C1)/I(H3) and G(C1)/H(H4) indicate that 1,3-β-Galp and 1,4-β-Galp are both connected to 1,3- and 1,4-α-Glcp at C3 and C4, respectively. Correlations A(H1)/E(C6), A(H1)/C(C3), and A(H1)/E(C3) show that T-α-Araf links to the C6 and C3 of 1,3,6-β-Galp and to the C3 of 1,3,5-α-Araf. Similarly, B(H1)/F(C6), B(H1)/G(C6), and B(H1)/M(C3) demonstrate that 1,5-α-Araf connects to the C6 of 1,4,6-β-Galp, 1,4-β-Galp, and the C3 of 1,3-β-Galp. Furthermore, H(H1)/I(C3) and I(H1)/H(C3) confirm mutual linkages between 1,4- and 1,3-α-Glcp. The C(H1)/E(C3) peak suggests bonding between 1,3,5-α-Araf and 1,3,6-β-Galp, while G(H1)/C(C5) and E(H1)/B(C5) indicate that 1,4-β-Galp and 1,3,6-β-Galp are linked to the C5 of 1,3,5- and 1,5-α-Araf, respectively. Finally, F(H1)/G(C4) and D(H1)/F(C4) confirm that 1,4,6-β-Galp is connected to 1,4-β-Galp, and that T-β-Galp is bonded to the C4 of 1,4,6-β-Galp. Comprehensive analysis of monosaccharide composition, methylation and NMR analyses, the proposed primary structure of CSP is illustrated in Figure 3D.

### 3.4 CSP inhibits the progression of diabetes in DM mice

At 72 h following alloxan administration, FBG levels in the model cohort were markedly elevated relative to the control cohort (*p* < 0.05, Figure 2B), confirming successful DM induction. Subsequently, the mice were randomly distributed into three cohorts: untreated model, CSP-treated, and METH-treated cohorts. From the first week of treatment, the model group exhibited marked body weight loss compared to controls (*p* < 0.05). In contrast, both CSP and METH treatment effectively suppressed weight loss in diabetic mice (*p* < 0.05, Figure 2C). Oral glucose tolerance tests revealed that model mice had impaired glucose handling, as evidenced by elevated glucose levels across time points (Figure 2D). Both CSP and METH improved glucose clearance. The AUC for glucose tolerance was elevated in the model cohort (*p* < 0.05) and reduced by CSP or METH treatment (*p* < 0.05, Figure 2E). TG, TC, LDL-C, and HDL-C levels were also assessed (Figures 4F–I). The model group exhibited pronounced increases in TC, TG, and LDL-C, accompanied by a marked reduction in HDL-C, relative to the control cohort (*p* < 0.05). Administration of CSP or METH lowered TC, TG, and LDL-C (*p* < 0.05). Although HDL-C levels showed an upward trend following CSP intervention, the increase was not statistically significant (*p* > 0.05).

**Figure 4:**
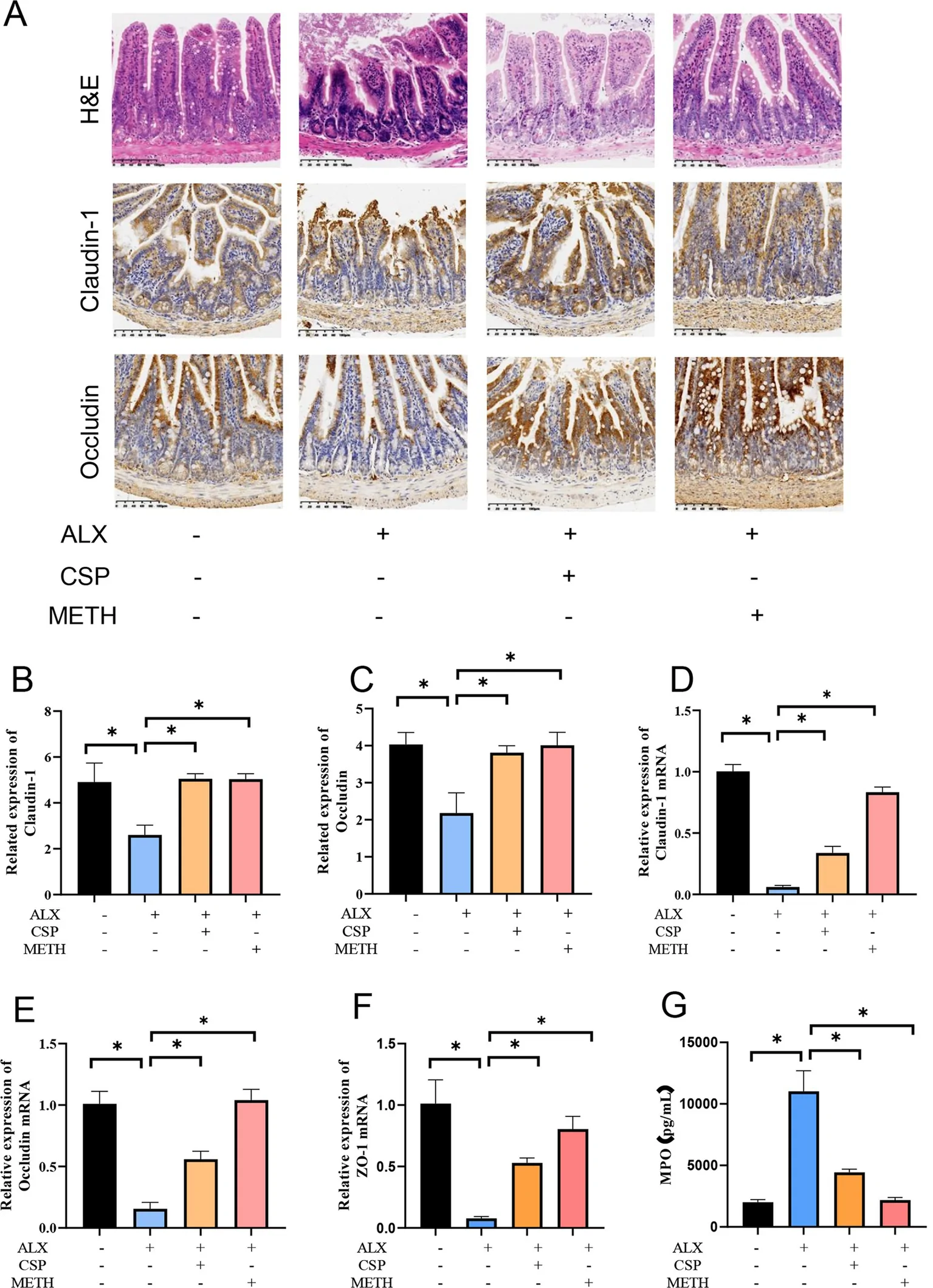
CSP promotes the repair of colonic mucosal injury in DM mice. **(A)** H&E staining and immunohistochemical analysis of Claudin-1, Occludin protein expression in colonic tissues of DM mice (magnification 400×; scale bar: 100 μm). **(B, C)** IHC quantification of Claudin-1, and Occludin protein expression levels in colonic tissues. **(D–F)** Relative mRNA expression levels of Claudin-1, Occludin, and ZO-1 in colonic tissues were determined by RT-PCR. **(G)** MPO levels in colonic tissues were measured by ELISA (*n* = 3; *p* < 0.05, *p* < 0.01 compared with model group).

### 3.5 CSP alleviates colonic injury in DM mice

Persistent hyperglycemia in diabetic patients impairs immune function and blood circulation, weakening host defense against infections. This disturbance promotes gut microbiota imbalance, elevating susceptibility to intestinal inflammation and UC (27). In addition, the gastrointestinal tract is critical in glucose homeostasis; UC can induce blood glucose fluctuations and exacerbate the progression of diabetes (28). Treatment with CSP or METH led to a notable downregulation of TNF-α, IL-1β, and IL-6 expression in colonic tissues (*p* < 0.05, Figure 5A). Immunohistochemistry and RT-PCR analyses confirmed that the protein (Figures 5B–D) and gene expression levels of TNF-α, IL-1β and IL-6 (Figures 5E–G) decreased significantly, indicating that CSP effectively inhibited intestinal inflammation in DM mice.

**Figure 5:**
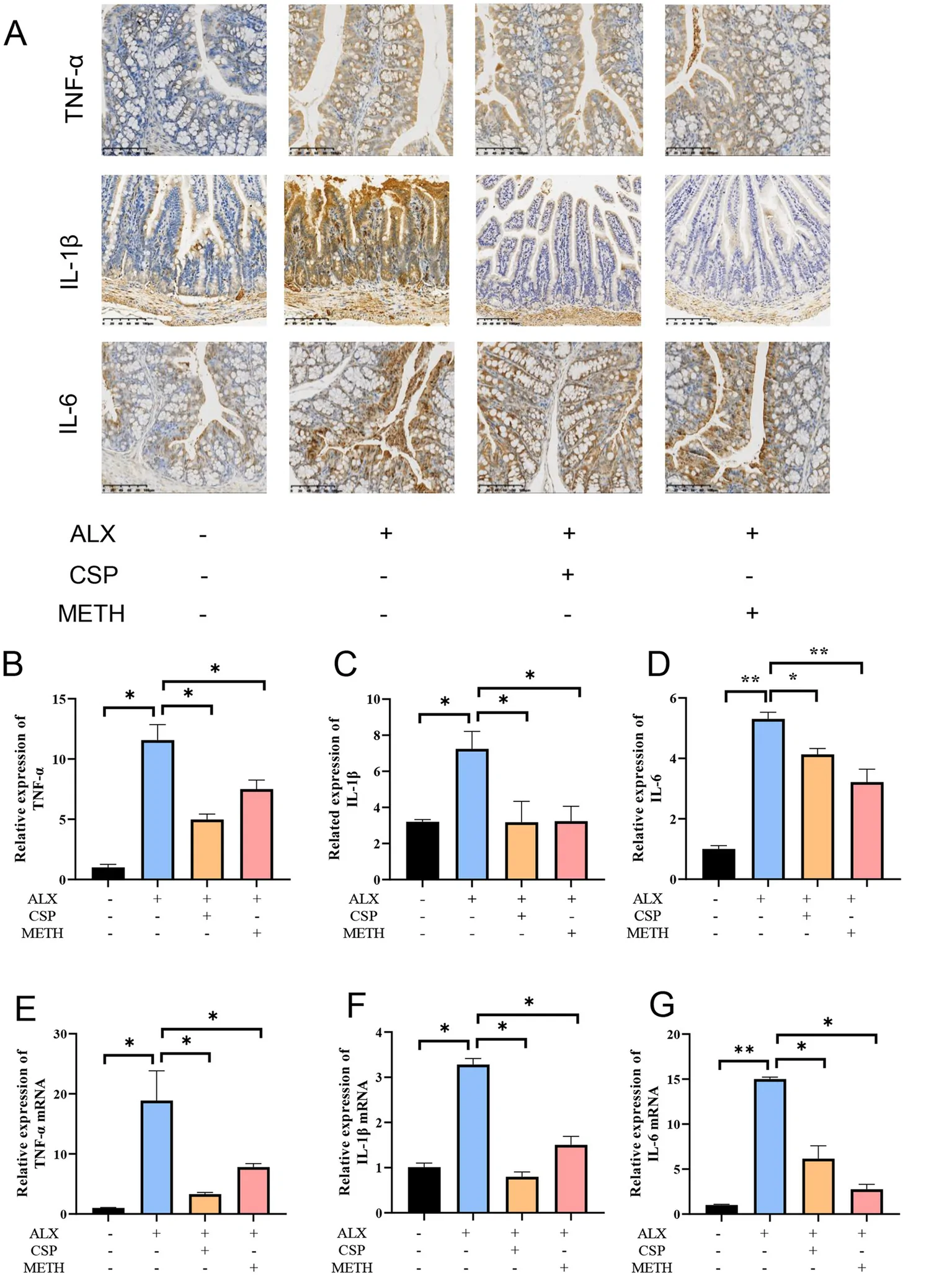
CSP attenuates colonic inflammatory injury in T1DM mice. **(A)** Immunohistochemical analysis of TNF-α, IL-1β, and IL-6 in colonic tissues of DM mice (magnification 400×; scale bar: 100 μm). **(B–D)** Protein expression levels of TNF-α, IL-1β, and IL-6 were detected by immunohistochemistry (IHC) in colonic tissues. **(E–G)** mRNA expression levels of TNF-α, IL-1β, and IL-6 in colonic tissues determined by RT-PCR (*n* = 3; *p* < 0.05, *p* < 0.01 compared with model group).

Histological analysis of DM mice revealed severe colonic mucosal disruption, including villus detachment, lamina propria exposure, necrosis, and inflammatory cell infiltration. Treatment with CSP or METH markedly attenuated mucosal damage, reducing villus edema and detachment (Figure 4A). Reestablishing intestinal barrier function is essential for colitis recovery. Tight junctions (TJs), formed by transmembrane proteins such as claudins, occludin, and zonula occludens-1 (ZO-1), constitute the core structural framework of the epithelial barrie (29). In DM mice, these TJ proteins were markedly reduced (*p* < 0.05, Figures 4B–F). Administration of CSP or METH significantly increased both gene and protein expression of these TJ-related proteins (*p* < 0.05). Myeloperoxidase (MPO), a lysosomal enzyme produced by neutrophils, reflects the extent of mucosal inflammation and neutrophil infiltration (30). MPO protein levels were significantly elevated in DM colonic tissue (*p* < 0.05, Figure 4G), whereas treatment with CSP or METH significantly decreased MPO levels (*p* < 0.05). CSP and METH demonstrated comparable efficacy in inhibiting the expression of pro-inflammatory cytokines (TNF-α, IL-1β, IL-6) in the colon, reducing MPO activity, and repairing intestinal tight junction proteins (Occludin, ZO-1). There was no statistically significant difference between the two groups (*p* > 0.05). These findings suggest that CSP improves DM-induced colonic injury by enhancing the expression of intestinal barrier-associated proteins and reducing neutrophil-mediated inflammation.

### 3.6 CSP ameliorates colonic injury by inhibiting NF-κB activation in DM mice

NF-κB functions as a central regulatory factor in the pathogenesis of ulcerative colitis, and its activation level correlates with the severity of intestinal inflammation (31). Previous findings demonstrated that CSP reduced TNF-α, IL-1β, and IL-6 expression in the colon of DM mice, suggesting a potential involvement of the NF-κB axis in CSP-mediated anti-inflammatory effects. To investigate the underlying mechanism, expression levels of proteins involved in the canonical NF-κB signaling axis were examined. As shown in Figures 6A,B, colonic tissues from DM mice showed marked increases in phosphorylated IκB (p-IκB) and phosphorylated NF-κB (p-NF-κB) (*p* < 0.05 and *p* < 0.01), suggesting pathway activation and enhanced inflammatory cytokine production. Following CSP treatment, the levels of p-IκB and p-p65 were markedly reduced (*p* < 0.05 and *p* < 0.01), reflecting effective inhibition of NF-κB activation. CSP inhibits the activation of the NF-κB pathway and the secretion levels of inflammatory factors TNF-α, IL-1β and IL-6, improves the repair level of colonic mucosa damage, and alleviates the diabetic ulcerative colitis in DM mice.

**Figure 6:**
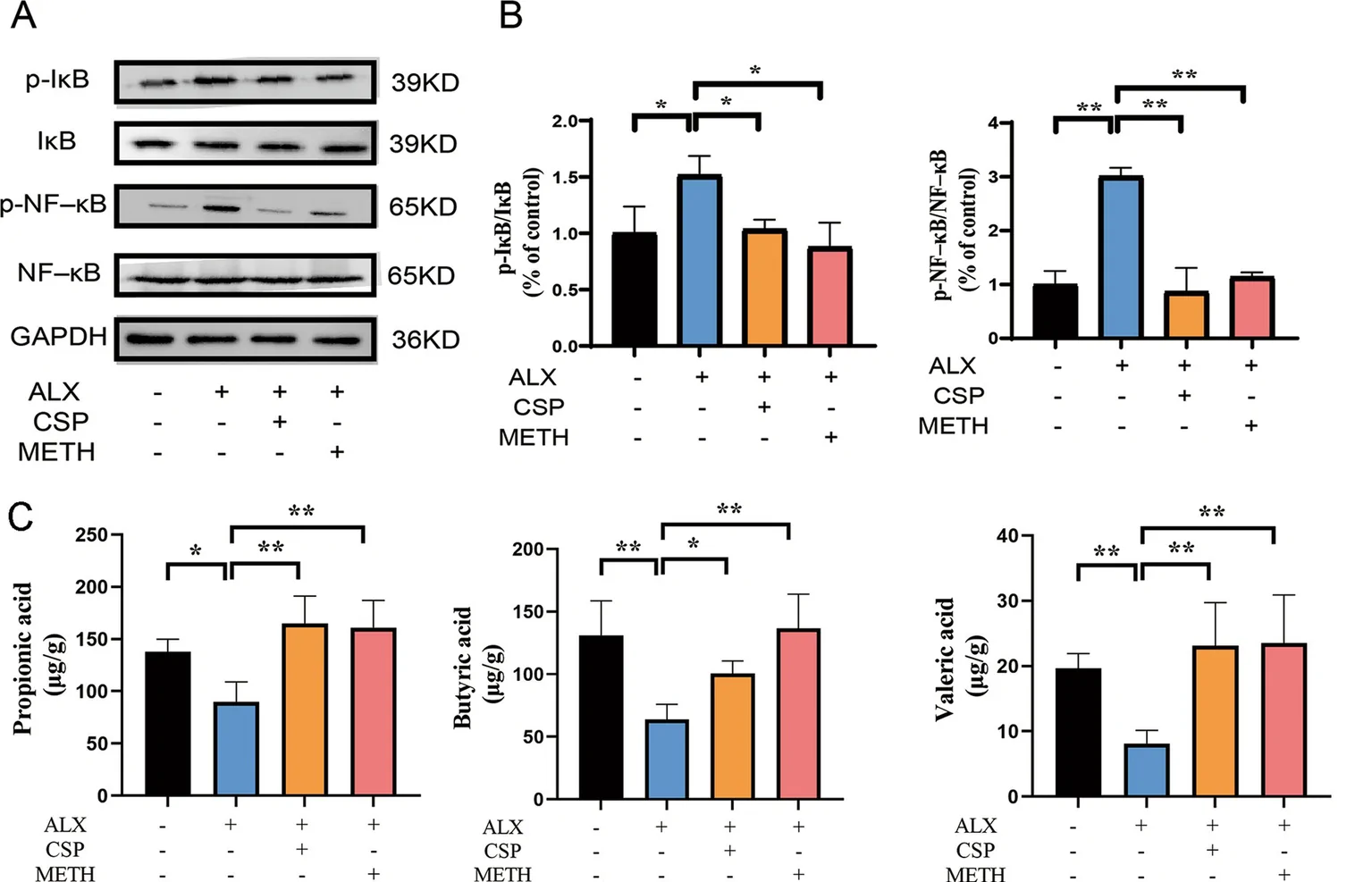
CSP alleviates colonic injury in DM mice by inhibiting NF-κB pathway activation and promoting SCFAs production. **(A, B)** In colonic tissues, western blot analysis of NF-κB pathway-related proteins, including p-IκB, IκB, p-NF-κB, and NF-κB (*n* = 3). **(C)** GC–MS quantification of SCFAs (propionic, butyric, and valeric acids) in colonic contents (*n* = 5) (*p* < 0.05, *p* < 0.01 compared with model group).

### 3.7 Effects of CSP on SCFAs profiles in DM mice

SCFAs, which are important metabolites of the intestinal microbiota, are relevant signaling molecules for diabetic ulcerative colitis. As shown in Figure 6C, the colonic contents of diabetic mice displayed significantly reduced levels of SCFAs, including propionic acid, butyric acid, and valeric acid (*p* < 0.05 and *p* < 0.01). In contrast, treatment with either CSP or METH markedly elevated the concentrations of these SCFAs (*p* < 0.05 and *p* < 0.01). Given their established roles in maintaining intestinal homeostasis and modulating inflammatory responses, the restoration of SCFAs levels by CSP and METH may contribute to their protective effects against diabetic ulcerative colitis.

## 4 Discussion

Shiliang tea has gained significant recognition primarily due to its notable antioxidant, anti-inflammatory, and antibacterial activities, particularly in the management of gastritis, UC, and metabolic syndrome. Its pharmacological effects have been attributed to various bioactive constituents, including volatile oils, coumarins, terpenoids, alkaloids, flavonoids, and quinones. Previous studies have demonstrated that volatile oils, flavonoids such as quercetin and kaempferol, and coumarin derivatives such as scopoletin exhibit potent antioxidant activity (1–3). Despite growing interest in Calycanthaceae phytochemicals, limited research has focused on the polysaccharides of this plant family. In the present study, we isolated and characterized a novel polysaccharide, designated CSP, from *Chimonanthus salicifolius*. CSP was identified as a neutral heteropolysaccharide mainly composed of Ara, Gal, and Glu, with a yield of 2.45 g/kg. The polysaccharides (COP) isolated from the plant of the same family, *Chimonanthus nitens* Oliv., contain Ara, Man, Glu and Gal (32), which is similar to the monosaccharide composition of CSP. Previous studies have shown that COP can alleviate acute hepatitis in mice by inhibiting pro-inflammatory cytokines (TNF-α, IL-1β and IL-6) (33), suggesting that the polysaccharides from the *Chimonanthus* may have potential value in the treatment of inflammatory-related diseases. Previous studies have shown that the extract of *Chimonanthus salicifolius* can exert an improving effect in ulcerative colitis models by regulating the intestinal flora and inhibiting the release of inflammatory factors (14). However, the impact of CSP on intestinal mucosal damage and intestinal barrier function related to diabetes remains unclear.

Polysaccharides are key bioactive components in traditional Chinese medicine and exhibit promising therapeutic effects on gut microbiota-related disorders, largely due to their indigestibility in the upper gastrointestinal tract. Upon reaching the colon, they are fermented by resident commensal microbes. This process enhances the growth of probiotic populations and results in the production of health-promoting metabolites. These effects are instrumental in alleviating conditions linked to gut microbial imbalance, such as diabetes, UC, and cancer (34–36). For instance, astragalus polysaccharides improve glycemic control by modulating insulin activity and its signaling pathways, thereby delaying diabetes progression. In addition, astragalus polysaccharides have demonstrated anti-inflammatory effects by reducing cytokine secretion and enhancing intestinal mucosal repair in DSS-induced colitis models (37, 38). *Lycium barbarum* polysaccharides exert hypoglycemic and insulin-sensitizing effects by enhancing antioxidant capacity, promoting glucose metabolism, increasing insulin secretion, and stimulating pancreatic β-cell proliferation. Moreover, Lycium polysaccharides alleviate DSS-induced colitis through the regulation of inflammatory cytokines and tight junction proteins (39, 40). Although numerous plant-derived polysaccharides have demonstrated therapeutic value, the efficacy of CSP in treating diabetes-associated ulcerative colitis remained unclear. In this study, CSP administration significantly decreased the area under the glucose tolerance curve in DM mice and modulated serum lipid profiles, including TG, TC, HDL-C, and LDL-C, indicating attenuation of diabetic progression. Given the documented link between DM and colitis development, it was hypothesized that CSP may alleviate colitis by mitigating the diabetic condition. The results show that CSP enhanced intestinal barrier integrity by upregulating Claudin-1, Occludin, and ZO-1. Additionally, CSP suppressed colonic levels of TNF-α, IL-1β, IL-6, and MPO, confirming its anti-inflammatory effects.

Aberrant cytokine production and dysregulated signal transduction in intestinal epithelial cells, lymphocytes, and macrophages contribute significantly to the pathogenesis of UC. Among these pathways, NF-κB plays a pivotal regulatory role, with its activation levels strongly correlating with the severity of intestinal inflammation (41). Dysregulated NF-κB axis contributes to chronic inflammation by impairing epithelial barrier function and disrupting crosstalk between the gut immune system and resident microbiota. In addition, activated NF-κB translocates into the nucleus, inducing the transcription of pro-inflammatory cytokines like TNF-α and IL-6, as well as chemokines, thereby promoting inflammation-related tissue injury (42, 43). Suppressing NF-κB axis is essential for alleviating colonic mucosal damage. Previous studies have reported protective effects in DSS-induced ulcerative colitis through inhibition of NF-κB activation and modulation of gut microbiota composition (44). Liu et al. indicated that blocking the NF-κB pathway reduced inflammation in DSS-treated rats (45). The outcomes of this investigation align with previously reported findings. Induction of DM colitis led to marked activation of the NF-κB axis, as evidenced by increased p-IκB and p-NF-κB levels, accompanied by elevated TNF-α, IL-1β, and IL-6, which aggravated mucosal inflammation. Administration of CSP significantly reduced p-IκB and p-NF-κB expression and inhibited the synthesis of these inflammatory mediators. To further investigate the effects of CSP on the intestinal metabolic environment, we measured the concentrations of selected SCFAs, including propionate, butyrate, and valerate. These metabolites have been implicated in the maintenance of intestinal homeostasis through their effects on epithelial barrier function, immune regulation, and host metabolism. In the present study, CSP administration significantly increased the levels of propionate, butyrate, and valerate that were reduced by STZ treatment. These findings suggest that CSP may help restore SCFAs-associated intestinal metabolic homeostasis in alloxan-treated mice. The above research indicates that CSP can inhibit the activation of the NF-κB pathway, reduce the secretion of inflammatory factors, improve intestinal epithelial damage in mice and the intestinal metabolic homeostasis related to SCFAs, and treat ulcerative colitis caused by TIDM.

This study has the following limitations: (1) higher-order conformational characterization of CSP was not performed, leaving its solution conformation and structure–activity relationship to be elucidated; (2) intestinal inflammation occurred in a diabetic background rather than classic colitis models, making it difficult to distinguish direct anti-inflammatory effects from indirect glucose-lowering effects, thus limiting causal inference; (3) this study did not directly observe the nuclear translocation of NF-κB. Subsequent research will further verify the crucial role of NF-κB in the anti-inflammatory effect of CSP through immunofluorescence staining and nuclear cytoplasm separation techniques; (4) the sample size was small (n = 5), affecting statistical robustness and generalizability; (5) 16S rRNA sequencing was not performed, precluding assessment of gut microbiota compositional changes and their potential mediating role in the observed anti-inflammatory effects. These will be addressed in future work using standard colitis models, conformational analyses, and larger sample sizes.

## 5 Conclusion

CSP, a newly characterized electrically neutral polysaccharide, was extracted and purified from *Chimonanthus salicifolius*, comprising arabinose, galactose, and glucose with a molar proportion of 4:5:10. Bioactivity studies demonstrated that CSP attenuated UC symptoms associated with diabetes in mice, largely via suppression of NF-κB pathway activation. CSP mitigated colonic inflammation by suppressing TNF-α, IL-1β, and IL-6, while promoting epithelial barrier repair through upregulation of Claudin-1, Occludin, and ZO-1. This study observed at the animal level that CSP has a mitigating effect on diabetes-related inflammatory indicators. This result provides preliminary experimental clues for the subsequent mechanism research of CSP.

## Data Availability

The raw data supporting the conclusions of this article will be made available by the authors, without undue reservation.
