# Supplementary material for: Structural characterization and effect on diabetes mellitus with ulcerative colitis of polysaccharide from Chimonanthus salicifolius
Source: Front Nutr. 2026 Sep 21;13:1934103. doi: 10.3389/fnut.2026.1934103 (PMC13635356; doi:10.3389/fnut.2026.1934103)
Supplement: Supplementary file 1 [file Data_Sheet_1.PDF]

Table S1 Primer sequences used for RT-PCR.

Gene	Forward primer	Reverse primer
IL-6-	CTTCTTGGGACTGATGCTGGTGAC	TCTGTTGGGAGTGGTATCCTCTGTG
IL-1β	TCGCAGCAGCACATCAACAAGAG	AGGTCCACGGGAAAGACACAGG
TNF-α	CGCTCTTCTGTCTACTGAACTTCGG	GTGGTTTGTGAGTGTGAGGGTCTG
ZO-1	GCGAACAGAAGGAGCGAGAAGAG	GCTTTGCGGGCTGACTGGAG
Occludin	TGGCTATGGAGGCGGCTATGG	AAGGAAGCGATGAAGCAGAAGGC
Claudin-1	GCTGGGTTTCATCCTGGCTTCTC	CCTGAGCGGTCACGATGTTGTC
GAPDH	GGCAAATTCAACGGCACAGTCAAG	TCGCTCCTGGAAGATGGTGATGG
