# Supplementary material for: Structural characterization and effect on diabetes mellitus with ulcerative colitis of polysaccharide from Chimonanthus salicifolius
Source: Front Nutr. 2026 Sep 21;13:1934103. doi: 10.3389/fnut.2026.1934103 (PMC13635356; doi:10.3389/fnut.2026.1934103)
Supplement: Supplementary file 2 [file Data_Sheet_2.PDF]

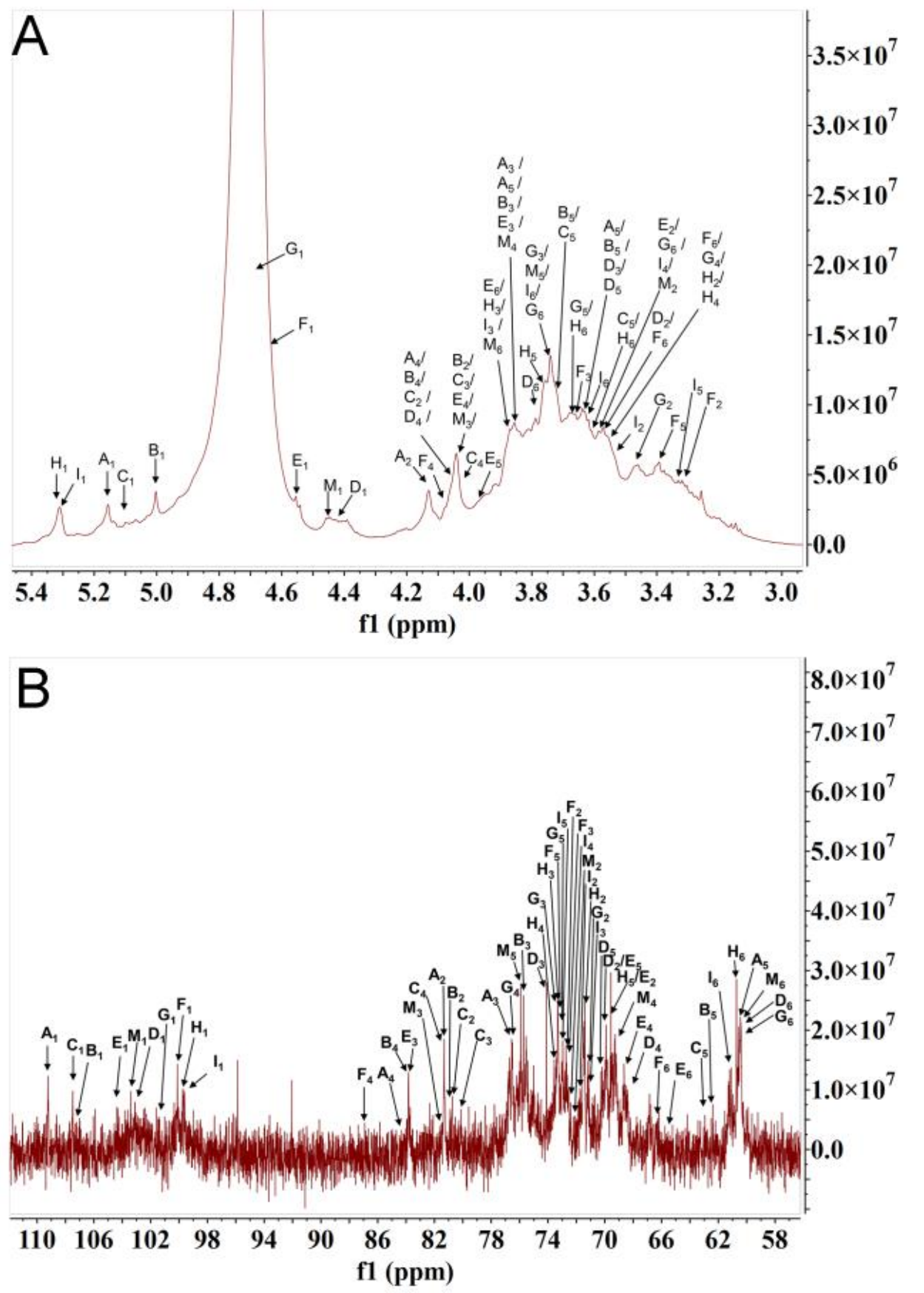

Figure S1. NMR analysis of CSP. (A) ^{1}H spectrum, (B) ^{13}C spectrum,

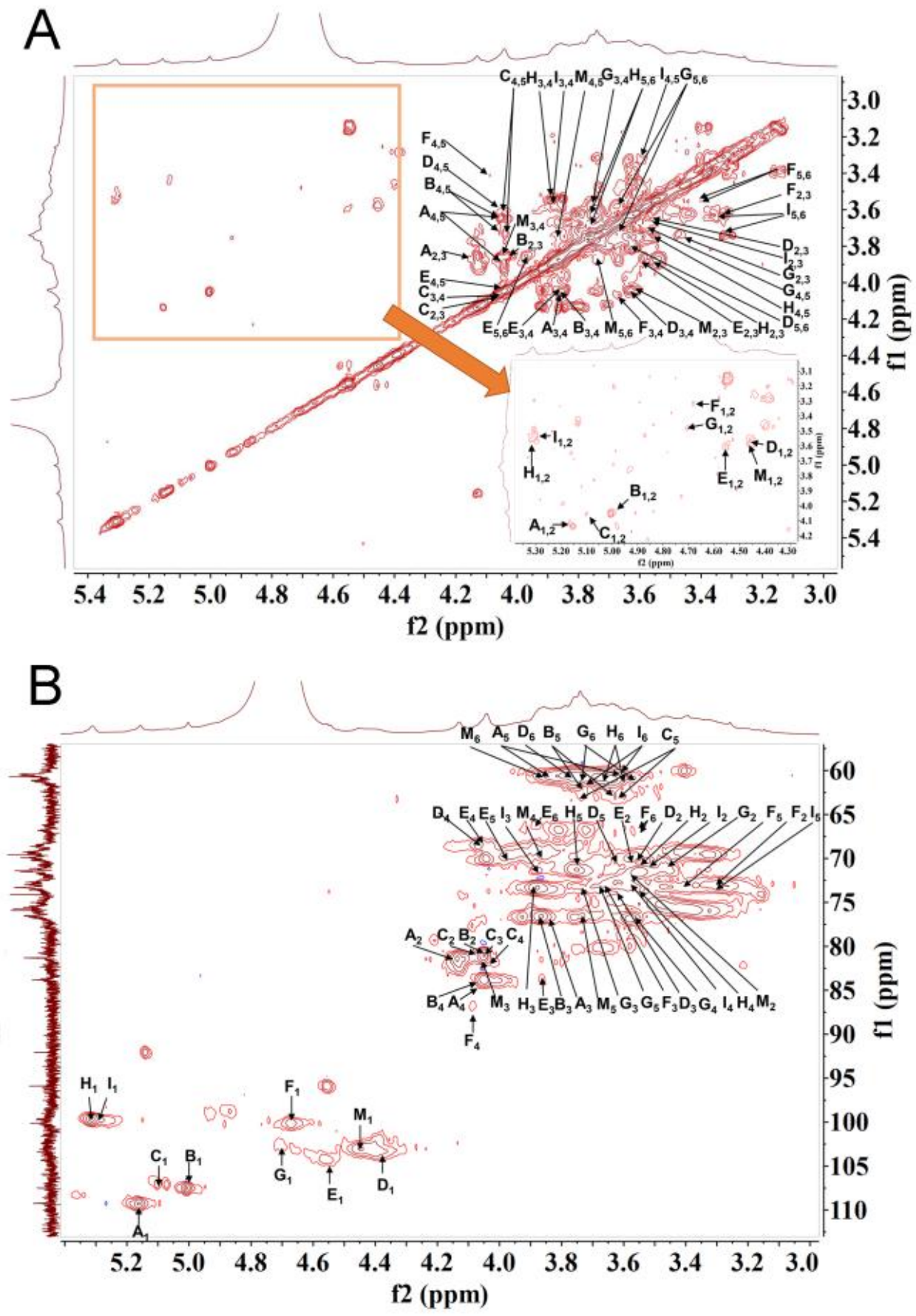

Figure S2. NMR analysis of CSP. (A) COSY spectrum, (B) HSQC spectrum.

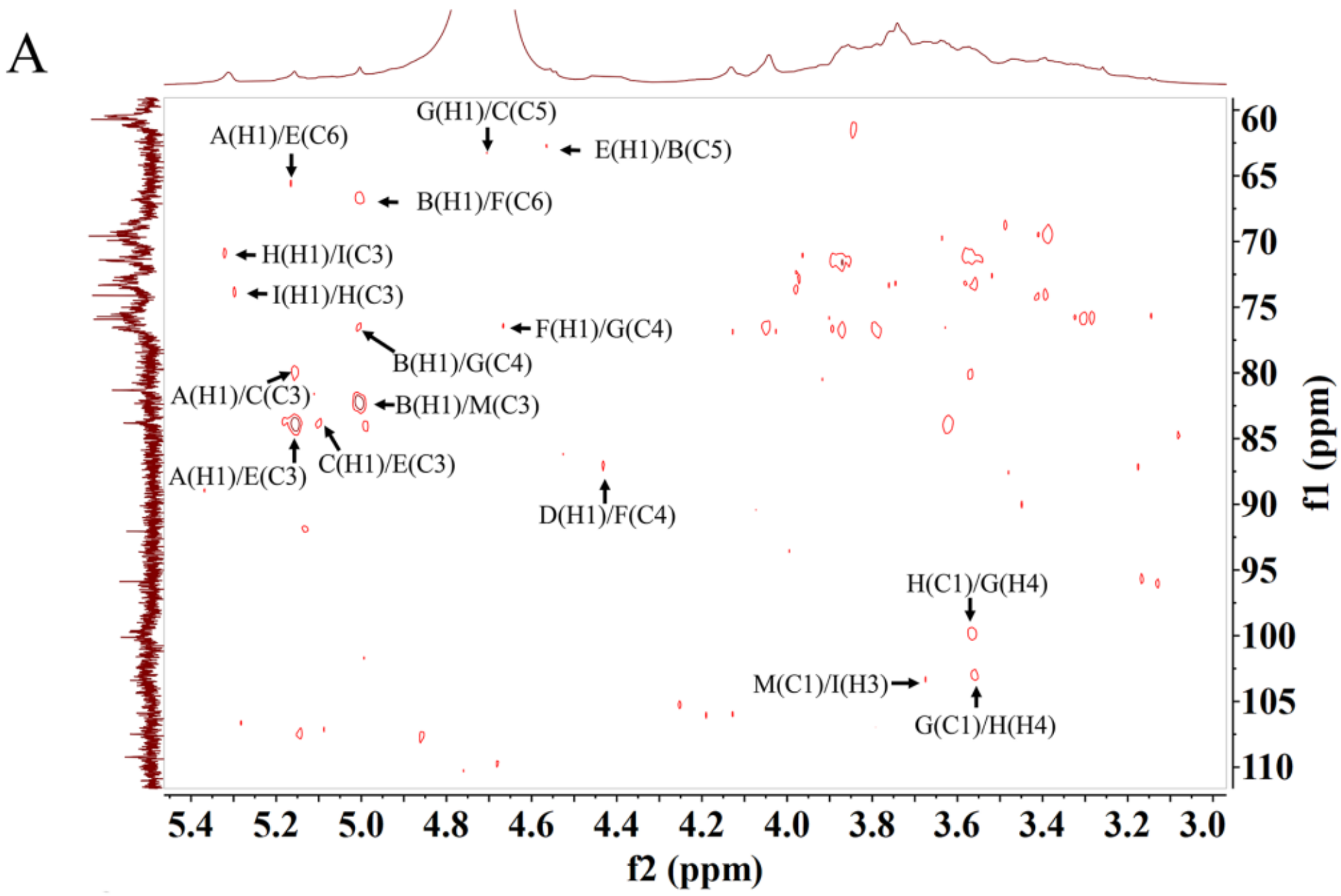

Figure S3. NMR analysis of CSP. HMBC spectrum
