# Supplementary material for: Structural characterization and effect on diabetes mellitus with ulcerative colitis of polysaccharide from Chimonanthus salicifolius
Source: Front Nutr. 2026 Sep 21;13:1934103. doi: 10.3389/fnut.2026.1934103 (PMC13635356; doi:10.3389/fnut.2026.1934103)
Supplement: Supplementary file 3 [file Data_Sheet_3.PDF]

Uncropped WB blot images for Figure 6A.

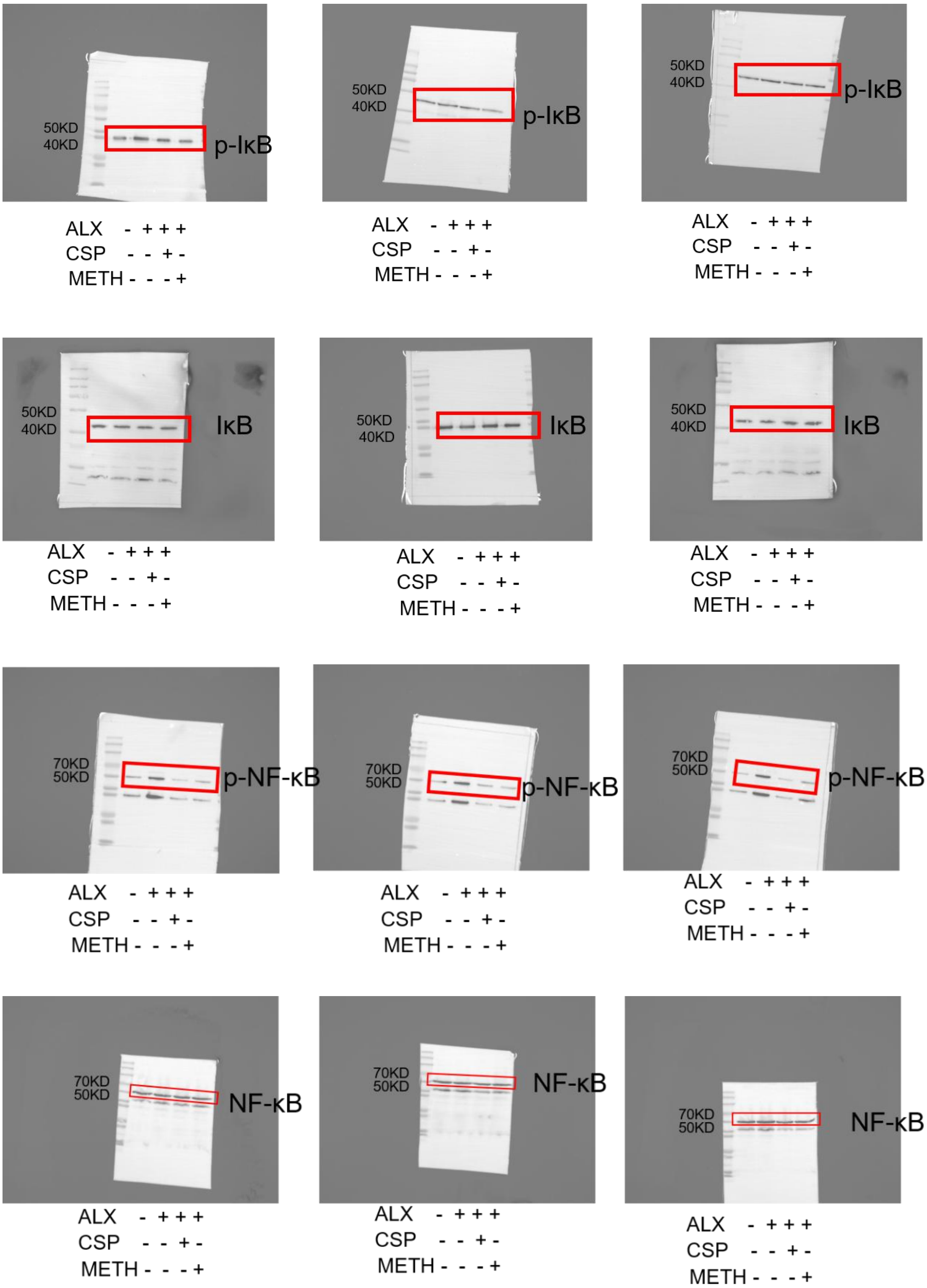

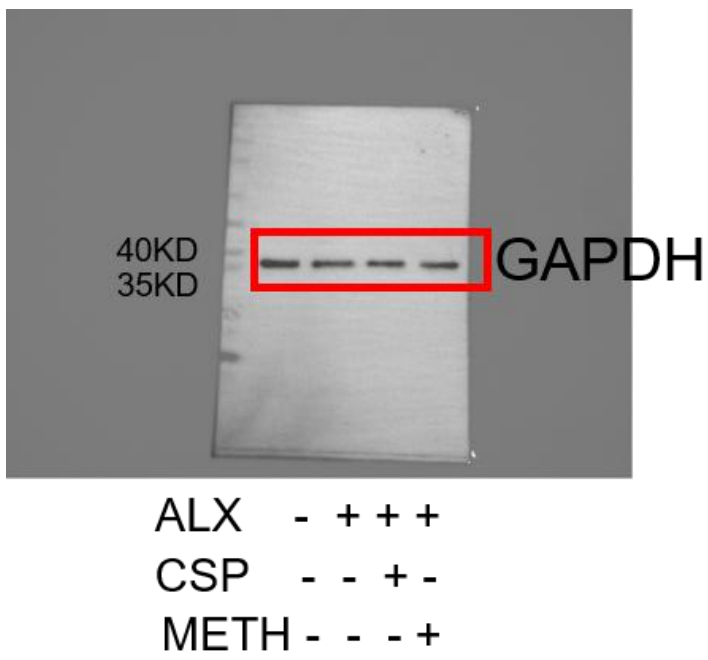
40KD
35KD
GAPDH
ALX - + + +
CSP - - + -
METH - - - +

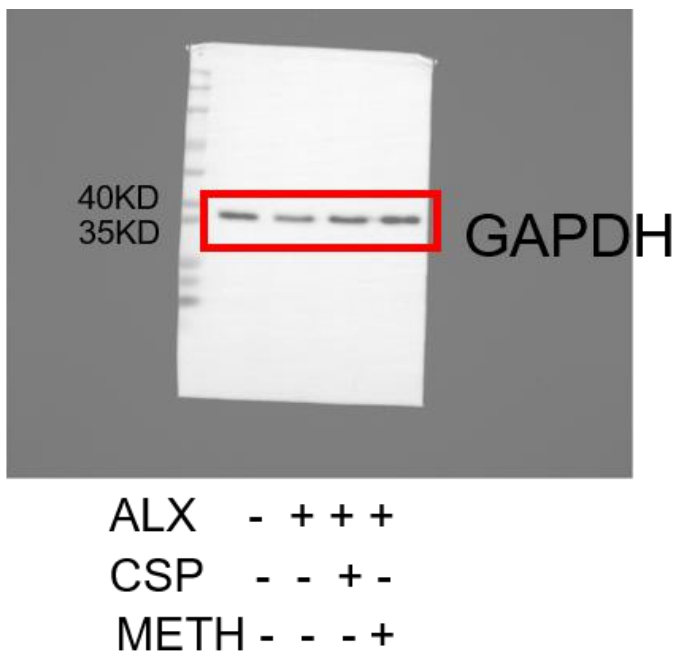
40KD
35KD
GAPDH
ALX - + + +
CSP - - + -
METH - - - +

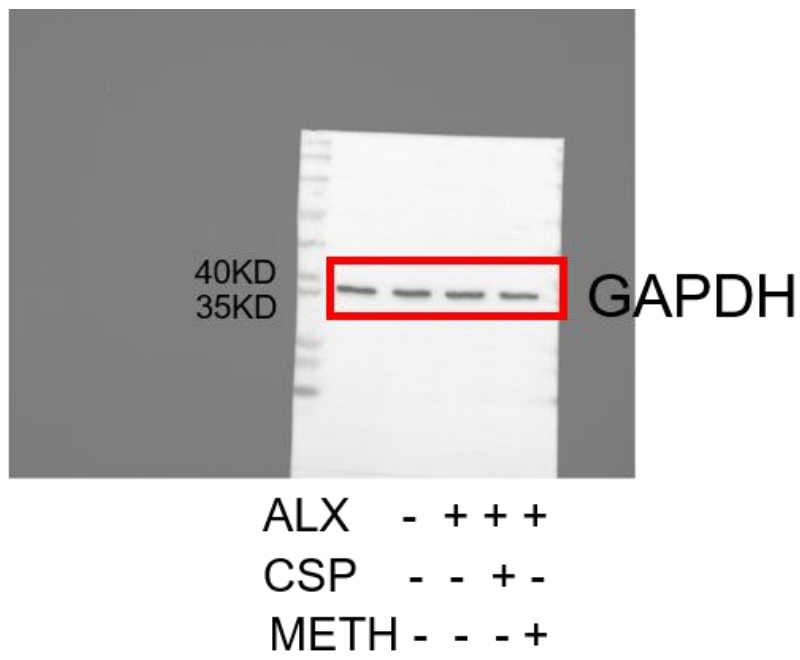
40KD
35KD
GAPDH
ALX - + + +
CSP - - + -
METH - - - +
